# ANFIS and ANN models to predict heliostat tracking errors

**DOI:** 10.1016/j.heliyon.2023.e12804

**Published:** 2023-01-05

**Authors:** Marie Pascaline Sarr, Ababacar Thiam, Biram Dieng

**Affiliations:** a Efficiency and Energetic Systems Research Group, Alioune Diop University of Bambey, Diourbel, Senegal; b Water, Energy, Environment and Industrial Processes Laboratory, Ecole Supérieure Polytechnique, Dakar, Senegal; c Renewable Energies, Materials and Laser Research Group, Alioune Diop University of Bambey, Diourbel, Senegal

**Keywords:** Heliostat tracking error, ANFIS, ANN, Solar tower power, Solar tracking

## Abstract

The efficiency and performance of solar tower power are greatly influenced by the heliostats field. To ensure accurate tracking of reflectors often requires an evaluation of the beam reflected positions. This operation is costly time-consuming due to the number of heliostats. It is also necessary to set up a fast and less expensive method able to evaluate tracking heliostat. In this paper, prediction models based on the Adaptive Neuro-Fuzzy Inference System (ANFIS) and Artificial Neural Network (ANN) were applied to estimate rapidly and accurately heliostat error tracking. The modeling is based on the experimental data of seven different days. The input parameters are time and day number and the output is the beam reflected position following the altitude and azimuth axes. Both techniques have been able to predict the beam reflected position. A comparison of results showed that intelligent methods recorded better performance than conventional model based on geometric errors. For ANFIS model, coefficients of correlation (R^2^) of 0.97 is obtained compared to that of the ANN, 0.96 and 0.92 for altitude and azimuth axes respectively. The intelligent methods may be a promising alternative for predicting heliostat beam reflected the position.

## Abbrivation

ANN: Artificial Neural Network
ANFIS: Adaptive Neuro-Fuzzy Inference System
DNI: Direct Normal Irradiance
MAE: Root Mean Squared Error
MF: Membership Function
MLR: Mcion
MSE: Mean Squared Error
RMSE: Root Mean Squared Error
R^2^: Coefficient of determination

## 1 Introduction

In solar tower power plants, the heliostat field represents 40–50% of the investment [1] and 40% of the annual energy losses [2]. An accurate heliostat tracking is essential for the optimal operation of solar tower power. Each heliostat tracks the sun individually and records its own errors. The relationship between the heliostat and its beam reflected position is very complex and depends on several parameters such as date, time and heliostat position.

Tracking errors are intrinsically random in nature and can occur due to several reasons. These errors generally involve atmospheric refraction, wind load-deformation, heliostat feedback, heliostat reflected beam spillover, pedestal tilt, azimuth and elevation reference, low resolution, and encoder type deformation of the reflecting surface, reduction of reflectivity, and reduction of sensor accuracy with aging [3].

To reduce tracking errors, it is necessary to define errors models for every heliostat.

Several models are based on geometric errors. This requires many measurements to find an accurate estimate of all model parameters for a particular heliostat [4]. This operation remains difficult, costly in time and money because of the number of heliostats.

An error correction method was implemented by Baheti and Scott [5] to reduce the mechanical errors associated to heliostat tracking. The errors are base tilt, azimuth and elevation bias offsets, and non-orthogonal drive axes. To estimate the tracking errors, a comparison is made between the commanded position and the real one. This allowed to obtain the error model coefficients that will be used to increase the tracking accuracy.

A study was conducted by Stone and Jones [6] to reduce the geometric errors of the heliostat tracking. To estimate these errors, an individual and sequential displacement of beam reflected towards a Lambertian target was done. A camera positioned on the ground makes it possible to quantify the error which is the deviation between target and beam reflected positions. These errors are then inserted at the level of the linear regression model.

Khalsa et al. [7] have extended the model of [5] at eight errors parameters on heliostat tracking. In this study, the offset between the reflected beam and the target was obtained using optical image analysis to calculate tracking errors.

A similar method was applied by Malan and Gauché [8] to reduce tracking errors by accounting for errors due to installation and manufacturing. The error model coefficients are obtained by quantifying periodically the tracking errors of each heliostat. Results show a reduction of open-loop tracking error to be inferior to 1 mrad.

The development of AI processes has allowed estimating researched data when the relation between output and input data is complex and not linear [9].

For Heliostat tracking, the use of AI techniques constitutes a real opportunity to address relevant challenges [10].

Among artificial intelligence techniques, ANFIS (Adaptive Nero-Fuzzy Inference System) and Artificial Neural Network (ANN) are described as powerful methods for predicting data [11,12]. Regardless of prior knowledge about the physical phenomenon and the nature of the relationships between the input/output variables, ANN and ANFIS networks can be used to interpret the behavior of complex nonlinear problems and, therefore, predict their future response. These two methods of prediction have been used in several fields [[13], [14], [15], [16], [17], [18]]. To determine the most suitable technique for predicting data, ANN and ANFIS performances have been compared in many instances [19,20].

In this paper, ANFIS and ANN have been used for heliostat tracking errors forecasting. The models' performance has been evaluated with the actual field data. To identify best model for every intelligent method (ANN and ANFIS), different effective parameters have been tested. Finally, a comparison study has been performed between the intelligent models predictions and the ones derived from the geometric model. The performance of these models was assessed through mean squared error (MAE), mean squared error (MSE), root mean squared error (RMSE), and coefficient of determination (R^2^).

## 2 Methodology

### 2.1 Experimental work and data collection

**Fig. 1:**
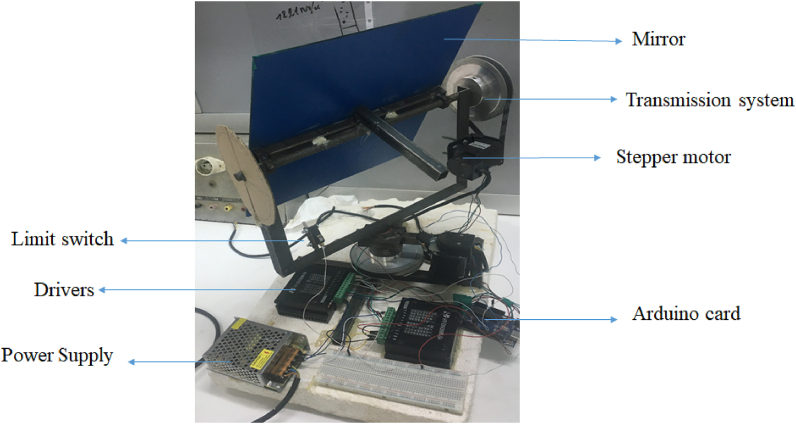
Mini heliostat prototype [22].

The study was performed using a mini heliostat prototype presented in Fig. 1. The experimental tests are done at Dakar, Senegal with a latitude of 14.7° N and a longitude of 17.4° W. To track the sun, a program based on the time, the number of day, and local coordinates is implemented.

**Fig. 2:**
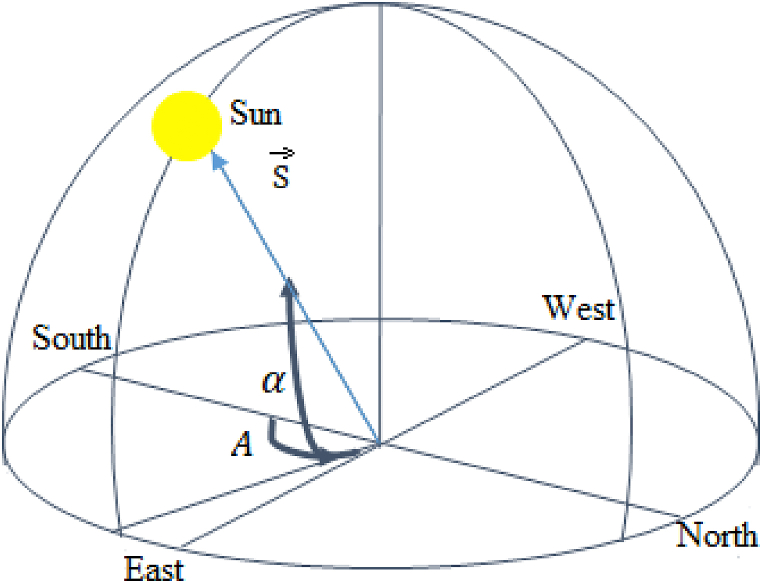
Illustration of sun angles.

Sun position $\vec{S}$ is located by solar azimuth A and solar altitude α as showed in Fig. 2 [23] and obtained from Eq. (1):

(1) $$\vec{S}=S_{z}\vec{i}+S_{e}\vec{j}+S_{n}\vec{k}$$

*S*_*z*_, *S*_*e*_ et *S*_*n*_ represent three orthogonal axes from the center of the earth pointing towards zenith, east and North, respectively. They are given by Eqs. (2), (3), (4)

(2) $$S_{z}=\sin\;\alpha$$

(3) $$S_{e}=\cos\;\alpha \;\cos\;A$$

(4) $$S_{n}=\cos\;\alpha \;\cos\;A$$

The solar altitude angle is the angle between the sun’s rays and the horizontal plane. The solar azimuth angle is the angular displacement between the projection of beam radiation on the horizontal plane and the South direction.

These positions vary according to the place latitude φ, the declination δ and the hour angle ω. They are given by Eq. (5) and (6):

(5) $$\alpha =\sin^{-1}\left(\sin\;\delta \;\sin\;\varphi +\cos\;\delta \;\cos\;\omega \;\cos\;\varphi \right)$$

(6) $$A=\cos^{-1}\left(\frac{\sin\;\delta \;\cos\;\varphi +\cos\;\delta \;\cos\;\omega \;\sin\;\varphi }{\cos\;\alpha }\right)$$

where *φ*, *δ* and *ω* respectively the latitude, declination and hour angle in degrees.

**Fig. 3:**
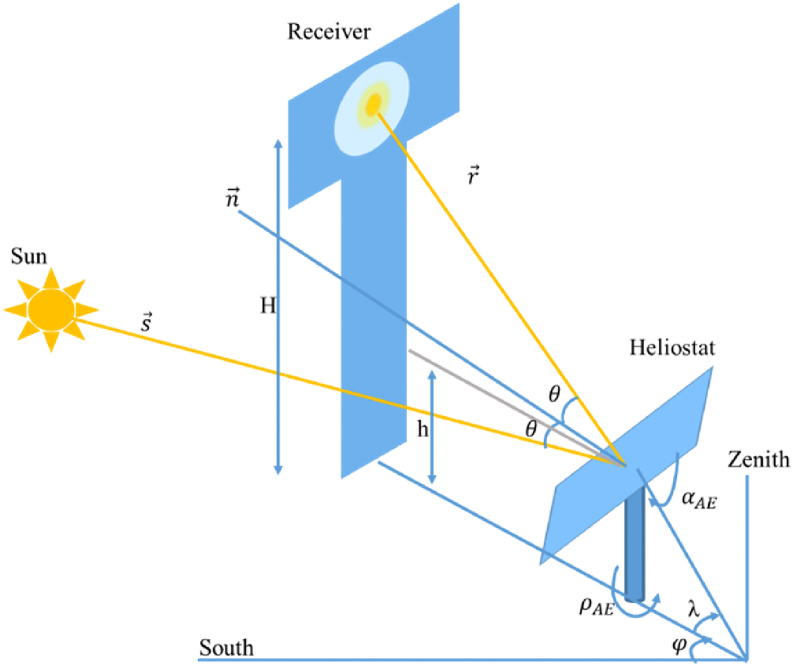
Illustration of heliostat tracking angles.

The normal vector of the heliostat $\vec{H}$ is defined by the altitude *α*_*AE*_ and azimuth *ρ*_*AE*_ angles. These angles, represented in Fig. 3, are determined from the sun vector position $\vec{S}$ and the target vector $\vec{r}$ and are calculated according to the following Eqs. (7), (8), (9)

(7) $$\vec{H}=\frac{\vec{S}+\vec{r}}{2\;\cos\;\theta }$$

(8) $$\alpha_{AE}=\sin^{-1}\left(\frac{\sin\;\lambda +\sin\;\alpha }{2\;\cos\;\theta }\right)$$

(9) $$\rho_{AE}=\sin^{-1}\left(\frac{\cos\;\alpha \;\sin\;A+\cos\;\lambda \;\sin\;\varphi }{2\;\cos\;\theta \;\cos\;\alpha_{AE}}\right)$$

where the different components are expressed in Eqs. (10), (11), (12), (13)

(10) $$\vec{r}=r_{z}\vec{i}+r_{e}\vec{j}+r_{n}\vec{k}$$

(11) $$r_{z}=\sin\;\lambda$$

(12) $$r_{e}=\cos\;\lambda \;\sin\;\varphi$$

(13) $$r_{n}=\cos\;\lambda \;\cos\;\varphi$$

*λ*, *φ*, *ϴ* respectively the focal, face and incidence angles in degrees.

**Fig. 4:**
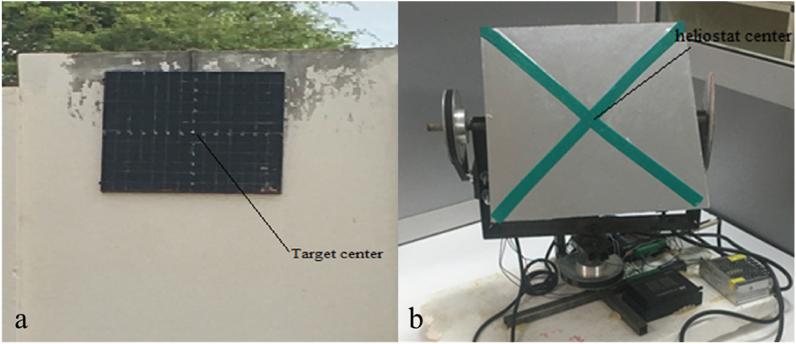
Target (a) and heliostat (b) centers identification.

**Fig. 5:**
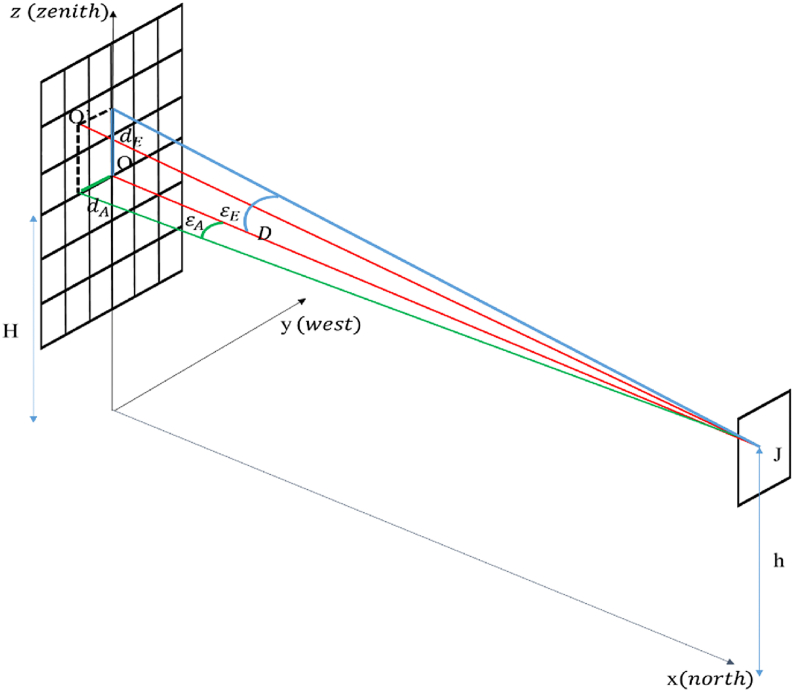
Heliostat tracking errors.

The error consists of a shift between the target point and the centroid of the reflected image. These tracking errors are quantified by making graduations of 5 cm on the target with center represented by the white point of coordinates (0, 0, H) as shown in Fig. 4a. The centroid of the reflected image is identified thanks to the diagonals realized on the mirror as illustrated in Fig. 4b. Error is quantified by reading the difference between beam reflected and target centers as shown in Fig. 5.

Tracking errors (altitude or azimuth) are given by Ref. [24] and expressed in Eqs. (14) and (15):

(14) $$\varepsilon_{A}=\mathrm{atan}\left(\frac{d_{A}}{D}\right)$$

(15) $$\varepsilon_{E}=\mathrm{atan}\left(\frac{d_{E}}{D}\right)$$

where.

*d*_*A*_ and *d*_*E*_ are respectively the azimuth and altitude errors in cm
*D* represents the distance between heliostat (J) and tower (O) centers obtained by the laser distance meter (m)
*O*′ is the reached point and h the heliostat height (m)

According to Al Rousan [25], employment of month, day, and time variables to predict solar tracking performance is better than using other variables. Date and time have great effects on beam reflected position on target [26]. In this study, the month and day are grouped in one variable, the number of day n (n = 1 for the 1st January and 365 for 31st December). The use of experimental data allows to set up rules in real time.

Experimental data of different days are used to train the neural network models. The number of the day and the time are selected as inputs data and the azimuth or altitude error constitute the output. Each tracking axis (azimuth or altitude) has its own training model.

### 2.2 Artificial neural network

**Fig. 6:**
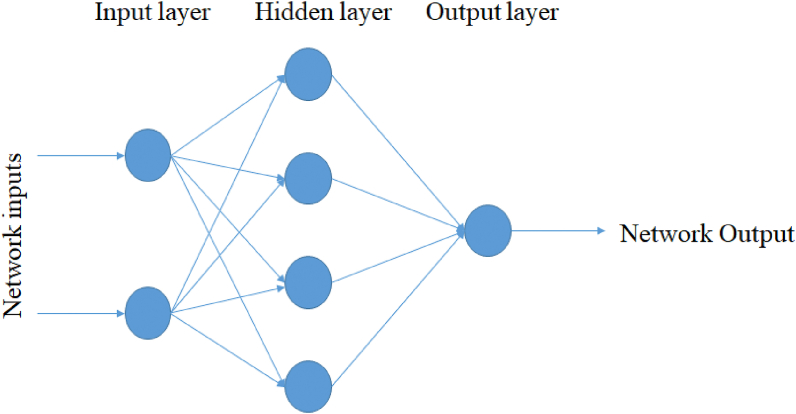
Schematic illustration of an artificial neural network with one hidden layer.

ANNs were established to imitate the architecture of the biological brain of humankind [27]. They are able to discover the nonlinear relationship in the input data set [28]. ANNs were used for the modeling, identification, and prediction of complex systems. They consist of input neuron layers, one or more hidden neuron layers, and a final layer which consists of the output neurons as illustrated in Fig. 6.

The optimum model is obtained by varying parameters of ANN training like number of hidden layers, number of neurons per hidden layer, number of training iterations, optimization algorithms, activation function. Previous studies have shown that a single hidden layer is sufficient to achieve good results, even for complex systems [29].

However, the number of hidden neurons is crucial to ensure the efficiency of ANN networks [30]. In this study, the optimal model is obtained by varying the number of neurons in the hidden layer. To evaluate the effect of hidden nodes number on the performance of ANN model, two different models are compared. The first one has ten hidden neurons and the second one has five hidden neurons both for azimuth and altitude axis. The optimal model is the one that presents the minimal difference between the predicted and real values. The activation function is an important parameter that can affect the output response and the accuracy of the calculation. In this work, tan sigmoid transfer function is used.

Different optimization algorithms are used for the training of ANNs among which Levenberg-Marquardt, Gradient Descendent, Gradient Decent with Momentum [31,32].

In this study, feedforward back propagation and Levenberg–Marquardt algorithm are used to train models [33,34]. Neural Network Toolbox OF Matlab was used to conduct the ANN modeling and evaluation.

### 2.3 Adaptive neuro-fuzzy inference system

**Fig. 7:**
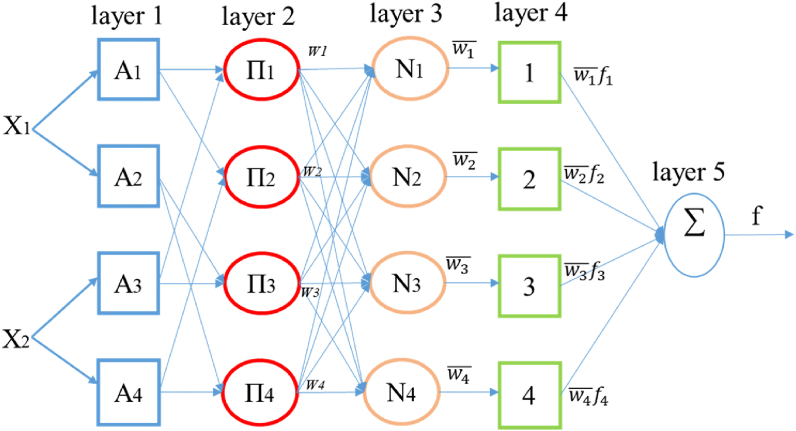
ANFIS system architecture.

ANFIS is a neuro-fuzzy technique that combines fuzzy logic theory and ANN learning laws. The structure of ANFIS consists of five layers, each containing several nodes described by the node function. An ANFIS model with two input parameters is presented in Fig. 7.

Layer 1 is the fuzzification layer. It receives the input data using membership functions and is described by Eq. (16) [35]:

(16) $$O_{i_{j}}=\mu_{A_{i}}\left(x_{j}\right)$$

where *i* denotes number of inputs, *x*_*i*_ is the input variable, *j* represents the number of membership functions for each input, and *μ*_*ji*_ is the MF which can be Gaussian, triangular, trapezoidal, and bell function.

At the layer 2 level, the logical operator AND is used to establish a link between the fuzzified inputs and the output of the layer as indicated by the following Eq. (17):

(17) $$w_{i}=\mu_{Ai}\left(x_{1}\right)*\mu_{Ai}\left(x_{2}\right)$$

where *i* = 1, 2 for *x*_*1*_ and *i* = 3, 4 for *x*_*2*_

Layer 3 allows normalization to obtain a normalized firing force. It is expressed in Eq. (18):

(18) $$\bar{w_{i}}=\frac{w_{i}}{w_{1}+w_{2}}$$

where *i* = 1, 2.

The normalized layer 3 output is then passed to the fourth layer. The output of each node in this layer is simply the product of the normalized firing power and a first order polynomial like expressed in Eq. (19).

(19) $$\bar{w_{i}}f_{i}=\bar{w_{i}}\left(p_{i}x_{1}+q_{i}x_{2}+r_{i}\right)$$

where in *p*_*i*_, *q*_*i*_, *r*_*i,*_ represent the if–then parameter rules, which are called consequent parameters. *fi* can be considered as output results and function of model and *x*_1_ and *x*_2_ are the model inputs Layer 5 has only one node. The sum of the signals coming from the previous layer is performed by Eq. (20)

(20) $$f=\sum_{i}\bar{w_{i}}f_{i}$$

*f* represent the total final output.

The optimized ANFIS architecture was selected based on the minimum residual error and the number of membership functions. The error of the output can be reduced by selecting the appropriate learning method. The performance of the ANFIS model depends greatly on the number and type of membership functions used [36].

In this study, the number of membership functions varies from 3 to 10. To verify the influence of MFs number on the model output, model with 15 membership functions is trained and compared to other models. The number of epochs is fixed at 500 for higher precision. For ANFIS network optimization, two methods are proposed namely back-propagation and hybrid which uses a combination of backpropagation and least-squares regression to tune the FIS parameters. In this study, the hybrid algorithm is chosen because of its fast convergence and accurate results [37]. For the Sugeno-type fuzzy system, output membership functions are either linear or constant. Both methods are studied to identify the optimal output MF according to the obtained results. By assumption, the Gaussian membership function is selected due to its higher prediction accuracy [38] and exact representation of the input-output relationship.

The mathematical representation of the Gaussian function is defined as Eq. (21):

(21) $$gaussian\left(x\right)={e^{-\frac{1}{2}\left(\frac{x-c}{\sigma }\right)}}^{2}$$

*c* and σ are respectively the mean and standard deviation

**Fig. 8:**
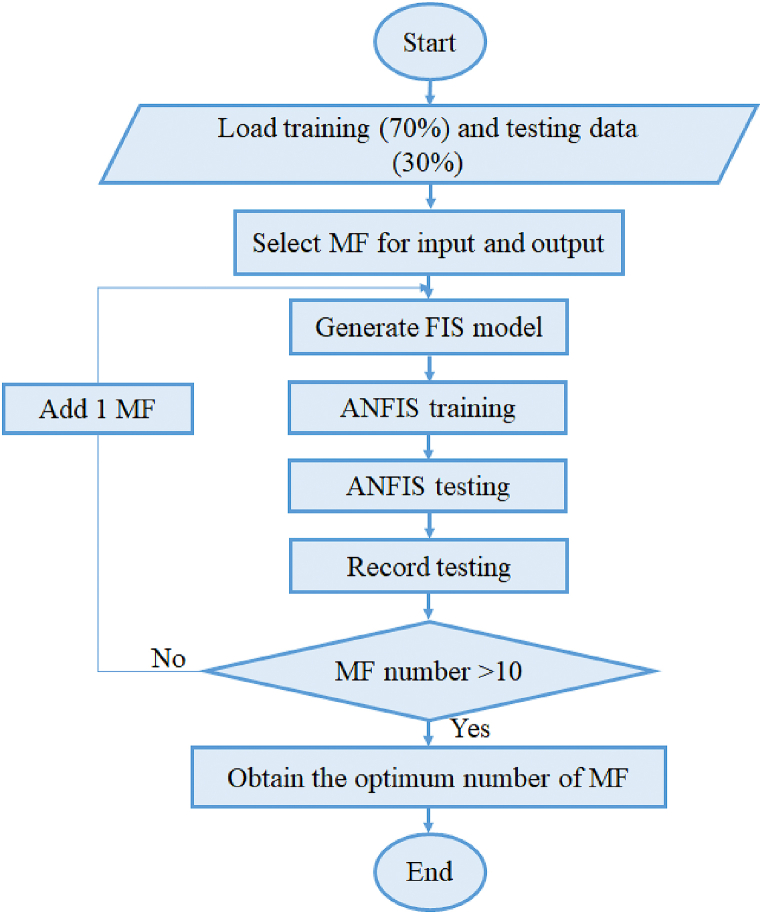
Flowchart of ANFIS

Fig. 8 illustrates the flowchart of ANFIS models system implemented in Fuzzy Logic ToolboxTM in MATLAB. For the data training, 70% of the experimental data is used while the remaining 30% is used for the testing.

### 2.4 Conventional error model

Conventional error model is derived as a function of heliostat position relative to the tower, solar vector, and the set of coefficients error geometry to predict the heliostat’s reflected beam offset on the target plane. In this study, the model established by Khalsa and al, [7] is used to predict heliostat beam position. The pointing error qi is given by Eq. (22):

(22) $$q_{i}=H_{i}.\varepsilon_{i}$$

where *q*_*i*_ is in function of two errors according to Eq. (23)

(23) $$q_{i}=\left(\begin{array}{l} d_{A}\\ d_{E} \end{array}\right)$$

*d*_*A*_ (*A*_*m*_ − *A*_*i*_) and d_E_ (*E*_*m*_ − *E*_*i*_) represent azimuthal and elevation errors respectively.

*A*_*i*_ and *A*_*m*_ are intended and measured azimuth angles, *E*_*i*_ and *E*_*m*_ the intended and measured elevation angles.

H_i_ defines a matrix function of pointing direction according to Eq. (24):

(24) $$H_{i}=\left(\begin{array}{cccccccc} \sin\;\theta \;\tan\;\alpha & \cos\;\theta \;\tan\;\alpha & 1 & 0 & \theta & 0 & \tan\;\alpha & \frac{1}{\cos\;\alpha }\\ \cos\;\theta & -\sin\;\theta & 0 & 1 & 0 & \alpha & 0 & 0 \end{array}\right)$$

Eq. (25) gives the different parameters of $\varepsilon_{i}$

(25) $$\varepsilon_{i}=\left(\begin{array}{c} \varepsilon_{1}\\ \varepsilon_{2}\\ \varepsilon_{3}\\ \varepsilon_{4}\\ \varepsilon_{5}\\ \varepsilon_{6}\\ \varepsilon_{7}\\ \varepsilon_{8} \end{array}\right)$$

*ε*_*i*_ vector assumed to be small, *ε*_1_, *ε*_2_ describe the pedestal tilt, *ε*_3_, *ε*_4_ the reference biases, *ε*_5_, *ε*_6_ linear errors, *ε*_7_ drive-axis non-orthogonality and *ε*_8_ boresight error.

The error parameter vector *ε* is estimated in Eq. (26) by using the least squares regression from a list of observations of *q*_*i*_ for various heliostat pointing angle:

(26) $$\varepsilon_{i}={\left[\sum_{i}H_{i}^{T}H_{i}\right]}^{-1}\left[\sum_{i}H_{i}^{T}q_{i}\right]$$

where *i* represents the total number of observations of azimuthal *d*_*A*_ and elevation *d*_*E*_ errors model.

To reduce the uncertainty of random errors, a great number of observations is necessary. Pointing error can be predicted for every position of heliostat by applying Eq. (22) after determining the error parameter vector.

### 2.5 Performance evaluation of models

To compare the different methods performance in relation to the experimental data, Eqs. (27), (28), (29), (30)) are used: mean absolute error (MAE), mean squared error (MSE), root mean square error (RMSE), coefficient of determination (*R*^2^).

(27) $$MAE=\frac{1}{n}\sum_{i=1}^{n}\left|l_{i}-y_{i}\right|$$

(28) $$MSE=\frac{1}{n}\sum_{i=1}^{n}{\left(l_{i}-y_{i}\right)}^{2}$$

(29) $$RMSE=\sqrt{\frac{1}{n}\sum {\left(l_{i}-y_{i}\right)}^{2}}$$

(30) $$R^{2}=\left(1-\frac{\sum_{i=1}^{n}{\left(y_{i}-l_{i}\right)}^{2}}{\sum_{i=1}^{n}{\left(y_{i}-\bar{y}\right)}^{2}}\right)$$

where *l*_*i*_, and *y*_*i*_ are respectively the predicted and true values; $\bar{y}$ the mean of predicted values and n is the number of samples.

## 3 Results and discussion

### 3.1 ANN prediction results

This section presents the results obtained with the ANN model. To obtain the best results, different ANN models have been evaluated. One of the most crucial modifications is the number of hidden nodes.

**Fig. 9:**
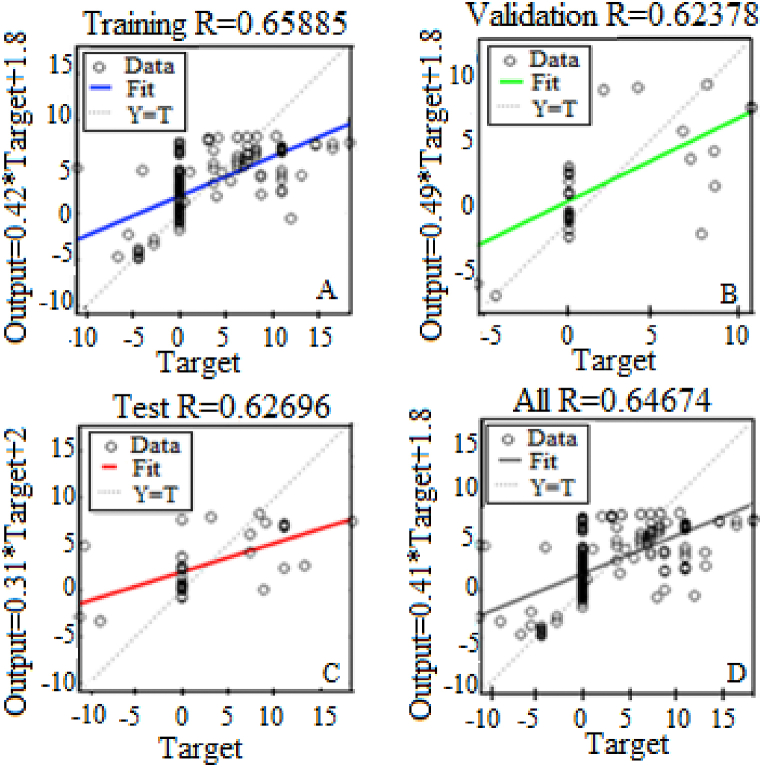
Results of the optimal model for altitude with 5 hidden neurons in training (A), validation (B), test (C) and All (D).

**Fig. 10:**
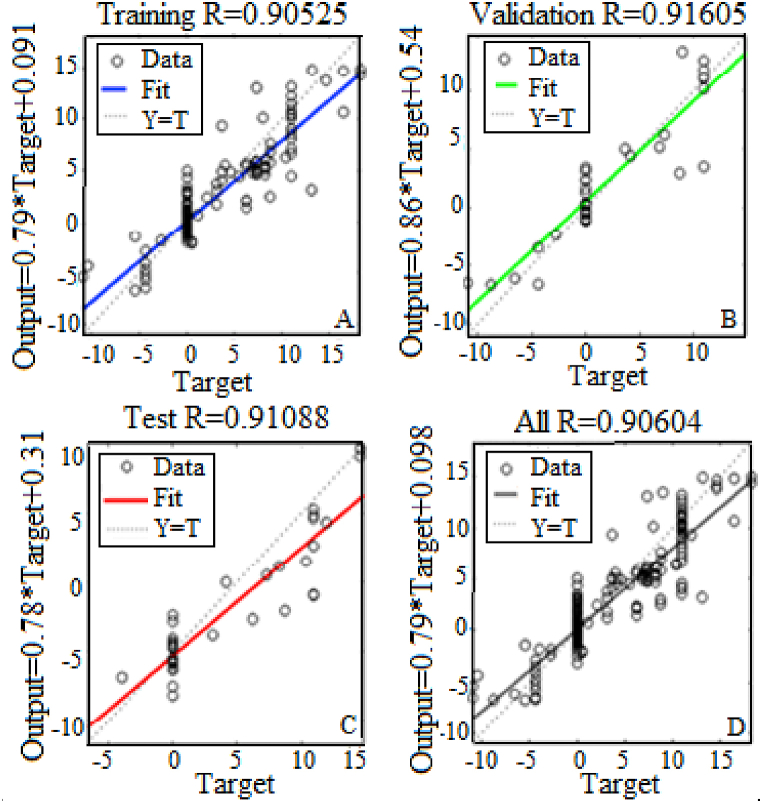
Results of the optimal model for altitude with 10 hidden neurons in training (A), validation (B), test (C) and All (D).

**Fig. 11:**
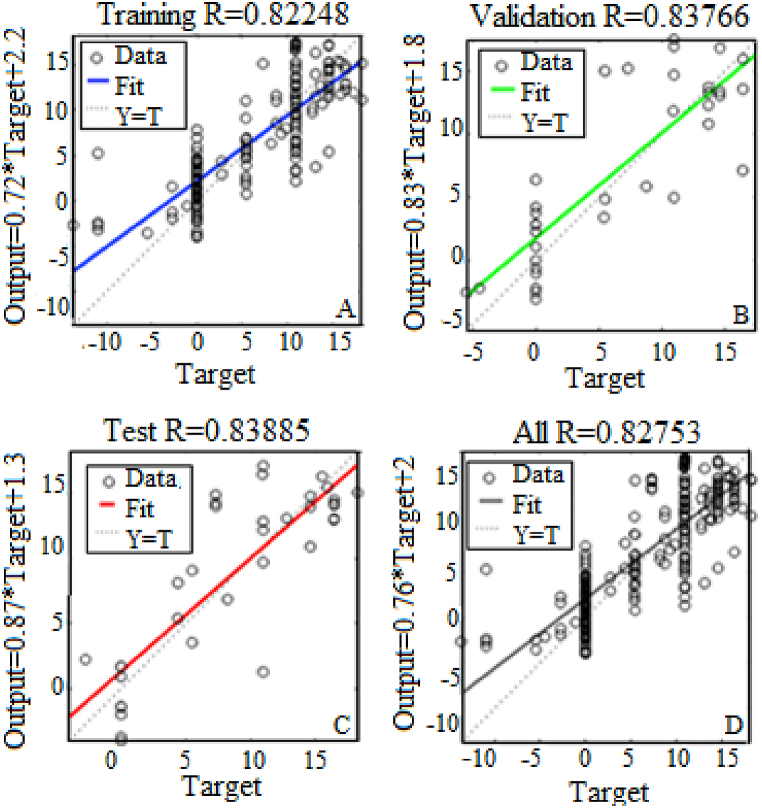
Results of the optimal model for azimuth with 5 hidden neurons in training (A), validation (B), test (C) and All (D).

**Fig. 12:**
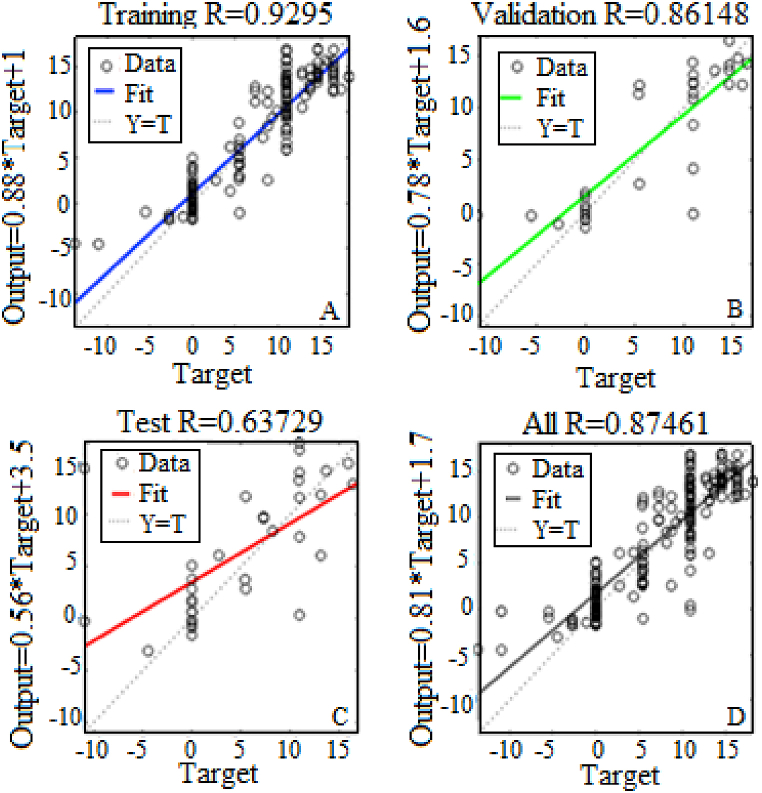
Results of the optimal model for azimuth with 10 hidden neurons in training (A), validation (B), test (C) and All (D).

Figs. 9–12 show that the best performance is obtained by using ten hidden nodes for the two axes. The models with ten hidden show the highest R-squared for training, validation, testing, and all data. Moreover, the data lie around a straight line passing a point close to the origin, which illustrates a very good agreement between the two. Training validation and testing show that the network learned from the data (R > 0.8). The fit is well aligned, showing that the model has good generalization and predictive ability.

### 3.2 ANFIS prediction results

To find the optimal model, two parameters are considered the number of mf and two MF output, linear or constant. Table 1 presents the learning errors of the different ANFIS models.

**Table 1:** Training error of different models.

Number of MFs	MF output	Altitude training error (mrad)	Azimuth training error (mrad)
(3,3)	Linear	0.164	0.192
(4.4)	Linear	0.160	0.154
(5,5)	Linear	0.137	0.145
(6,6)	Linear	0.130	0.133
(7,7)	Linear	0.11	0.123
(8,8)	Linear	0.120	0.130
(9,9)	Linear	0.075	0.108
(10,10)	Linear	0.063	0.085
(15,15)	Linear	0.045	0.05
(3,3)	Constant	0.234	0.223
(4.4)	Constant	0.197	0.19
(5,5)	Constant	0.174	0.184
(6,6)	Constant	0.177	0.153
(7,7)	Constant	0.165	0.145
(8,8)	Constant	0.139	0.143
(9,9)	Constant	0.154	0.135
(10,10)	Constant	0.143	0.130
(15,15)	Constant	0.109	0.101

The best results are obtained with the linear MF output models. Gaussian membership function will be adopted to represent input variables, while linear membership function will be used to represent the output variables. Among the linear models, the model (15,15) is optimal with smaller learning errors, 0.045 and 0.05 respectively for the altitude and azimuth axes, compared to the other models. This model will be considered for the comparative study in the next section.

### 3.3 Comparison between ANN, ANFIS and geometrical models

The comparison based on the statistical coefficients between experimental and models predicted values are presented in Table 2. All three models are able to predict heliostat beam reflected position on the target following two axes.

**Table 2:** Statistical coefficients of three models.

	Altitude			Azimuth		
	ANN	ANFIS	Conventional model	ANN	ANFIS	Conventional model
MSE (mrad)	0.06	0.028	0.16	0.134	0.037	0.208
MAE (mrad)	1.231	0.453	2.966	1.802	0.579	3.105
RMSE (mrad)	0.244	0.167	0.4	0.366	0.192	0.456
R^2^	0.964	0.973	0.89	0.925	0.97	0.87

The MSE between the predicted and experimental values for altitude and azimuth axes were found to be 0.028 and 0.037 for ANFIS, 0.06 and 0.134 for ANN and 0.16 and 0.208 for geometrical model. For MAE, the worst results are obtained with conventional method, 2.96 and 3.1, and the best results with ANFIS, 0.453 and 0.579, respectively for altitude and azimuth axes. For determination coefficient and Root Mean Square Error, ANFIS model presents the best results with highest R^2^ of 0.97 and lowest RMSE of 0.167 and 0.192 for altitude and azimuth axes respectively. The ANN model indicates slightly lower performance with R^2^ 0.964 and 0.925; RMSE of 0.244 and 0.366 for altitude and azimuth axes respectively. Conventional model presents inferior performances with a correlation coefficient of 0.8 and RMSE of 0.4 and 0.456. The results reflect a good fit between the predicted and experimental data, indicating the models suitability. ANFIS model performs better than the other models. Moreover, we noticed that best results are obtained with the elevation axis. This difference is due to the fact that wind effects are more important at the level of the azimuthal axis, hence presence of these numerous variations.

**Fig. 13:**
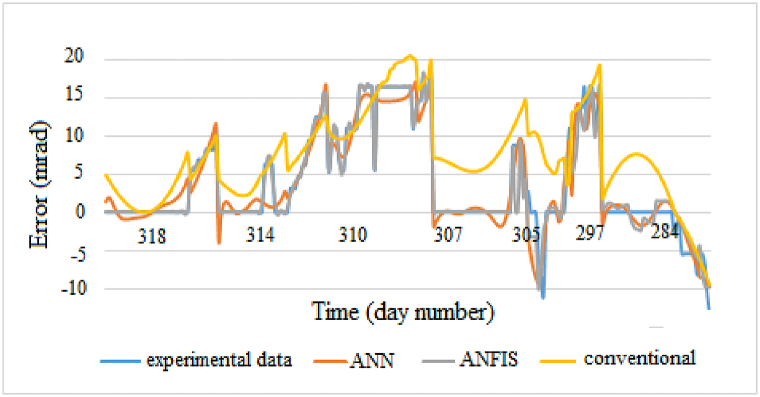
Altitude errors.

**Fig. 14:**
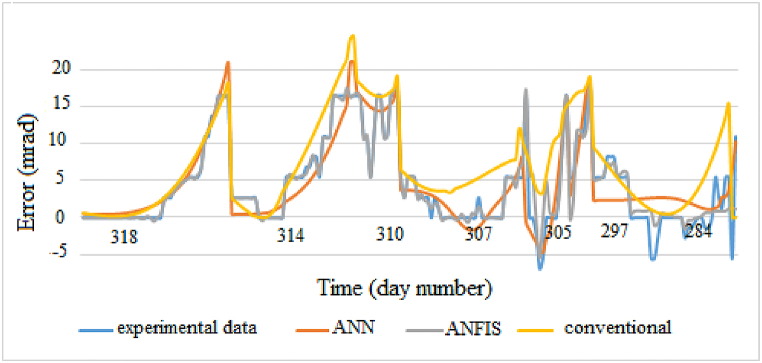
Azimuth errors.

Figs. 13 and 14 show the behavior of three models with the experimental data. Good performances are obtained for both intelligent models.

It is evident from these figures that the experimental and predicted ANFIS values are in close agreement compared to other methods. ANN model follows the trend of the experimental data but not with high accuracy. Geometric model describes the experimental data variation but is not efficiency with fluctuations. This is due to the fact that the conventional error model only takes into account geometrical errors not systematic errors such as wind effects can reduce the accuracy of this error model.

During the experiments, we noticed that neurons performance depend on the training data. On Figs. 13 and 14, a good agreement is noted at the beginning between predicted and experimental values. Towards the end, there is a disproportion between the two types of data. The negative values being more present in test data than training data, the system does not recognize these type of data. The use of fairly large errors will allow the system to adapt in case of extreme deviation of the reflected beam. The results indicate that the selection of data sets for training ANFIS models is an important factor affecting performance. The performance of the ANFIS system can also be improved by using different training data sets. The performance of a model also depends on the difference between training and test data. Larger is the difference, lower is the performance.

Best results are obtained with the elevation axis. This difference is due to the fact that wind effects on the heliostat are more important at the level of the azimuthal axis, hence presence of these numerous variations.

Compared to the previous methods, intelligent methods are easier to implement for complex systems and they are time-consuming cost. Classical methods require more materials, therefore, are more expensive. With conventional methods, the heliostats tracking is evaluated one by one, which takes more time.

To obtain the results similar to those in the article, the following steps must be respected. First, the model must be created by defining the databases, input and output values and choosing the fuzzy sets and rules as defined in this article. After, the model must be trained. This step consists in training the model and modifying model parameters until the best model is obtained. These parameters concern the number of hidden neurons for the ANN and the type of MFs for the ANFIS. The last step consists to evaluate models performance with new data.

Unlike classical models, intelligent systems do not have control function at the end of the training that characterizes the model in question.

The best model is chosen based on the difference between the predicted and experimental values. This model will be also able to predict the tracking errors of the heliostats at any time and day. Intelligent methods allow to predict the tracking errors deterministic and stochastic. The tracking errors correction become more easy.

One of the limitations of this study is the sun position. Indeed, the day duration and the position of the sun vary according to the seasons. The days are longer in summer and shorter in winter. However, the tests were carried out in October and November where the characteristics are different. Tests must then be carried out for the other seasons for having annual data. The other aspect is the position of the heliostat. The position is different from one heliostat to another. Another input taking into account the position of the heliostat can thus be added to the model.

## 4 Conclusion

In this paper, comparative study between intelligent and conventional models was realized to predict heliostat tracking errors. Time and day number were chosen as input parameters for modeling. To identify the optimal model for every artificial intelligence method, different models with various parameters were developed. The results showed that both techniques are very suitable to predict heliostat tracking errors but the ANFIS technique is better.

Therefore, ANFIS can be used to predict heliostat tracking errors with a high accuracy. The main advantage of intelligent techniques in comparison with classical methods is the reduction of the cost and time to evaluate heliostats beam reflected. It becomes easier to develop models of errors correction for the heliostats field. Furthermore, developed models can be improved by adding the heliostat position in the field as input.

[21]
